# An evolutionary model and classification scheme for nephrite jade based on veining, fabric development, and the role of dissolution–precipitation

**DOI:** 10.1038/s41598-022-11560-7

**Published:** 2022-05-12

**Authors:** Matthew S. Tarling, Steven A. F. Smith, Marianne Negrini, Li-Wei Kuo, Wei-Hsin Wu, Alan F. Cooper

**Affiliations:** 1 Department of Geology, University of Otago, Dunedin, New Zealand; 2 Department of Earth and Planetary Sciences, McGill University, Montréal, QC Canada; 3 Department of Earth Sciences, National Central University, Taoyüan, Taiwan; 4 Earthquake-Disaster and Risk Evaluation and Management Center, National Central University, Taoyüan, Taiwan

**Keywords:** Solid Earth sciences, Mineralogy, Tectonics

## Abstract

Although nephrite jade has been collected and treasured since the Stone Age, we lack a clear understanding of how it forms during deformation and metasomatism in shear zones. Using microstructural analysis of samples from Taiwan, California, and New Zealand, we propose a conceptual model for the evolution of nephrite jade that distinguishes four nephrite types based on mode of formation and textural characteristics: (1) primary (type 1a) or folded (type 1b) vein nephrite, (2) crenulated nephrite (type 2), (3) foliated semi-nephrite (type 3), and (4) nodular or domainal nephrite (type 4). We interpret the texture of our analysed samples to represent snapshots of a progressive textural evolution similar to that experienced by other deformed and fine-grained metamorphic rocks that develop under fluid-present, greenschist-facies conditions. Our observations suggest that types 2 and 3 nephrite can evolve from vein nephrite (type 1) by the development of crenulated and foliated metamorphic fabrics, during which the most important deformation process is dissolution–precipitation. However, development of metamorphic fabrics can be interrupted by transient brittle deformation, leading to the formation of type 4 nephrite that is characterised by nodular or angular clasts of nephrite in a nephritic matrix.

## Introduction

Nephrite jade is a monomineralic and microcrystalline rock dominated by the tremolite-actinolite series of amphiboles (Ca_2_(Mg,Fe)_5_Si_8_O_22_(OH)_2_). Nephrite derived from fluid-mediated metasomatic reactions involving serpentinite (as opposed to the less common dolomite-derived nephrite) occurs in orogenic belts worldwide (e.g., Canada, China, Russia, Taiwan, New Zealand, Australia, Poland, Italy), where it has been collected since the Stone Age as a gemstone and for use in tool- and weapon-making^1^. Nephrite is valued for its extreme fracture toughness, striking colour range, and semi-translucency: characteristics that relate to the texture and crystallography of the constituent amphibole aggregates^2–5^.

Previous studies recognised that nephrite is formed by metasomatic reactions within or along the margins of shear zones^1,6–10^. Reactions are common at the contacts between calcium-bearing lithologies and serpentinite, and involve the metasomatic addition of calcium and silica to serpentinite, following a general reaction such as 5 Serpentine + 14SiO_2_ + 6 CaO = 3 Tremolite + 7 H_2_O^1,6,8,11^. Nephrite is typically formed at low- to mid-greenschist facies, although examples range from ~ 100° to 550 °C and from ~ 0.1 to > 1 GPa^1,6,8,11^. Similar metasomatic reactions that occur outside shear zones or are post-kinematic result in coarser crystal sizes (mm-cm) and the formation of zoned reaction rinds^12–18^.

Classic petrological studies of nephrite report textures consisting of randomly-oriented bundles of “matted”, “felted” and “interwoven” fibres, in which individual crystals may be “twisted”^19–21^. The ubiquitous association between metasomatism and deformation during the formation of nephrite is noted in nearly all studies of serpentinite-associated nephrite^1,8–10,19–30^. Many of these studies broadly related the development of the typical nephritic texture to a combination of mineral reactions, shearing and mechanical processes. Turner (1935) recognised “intense shearing and mechanical breakdown” accompanied by “chemical reconstitution and growth of fresh crystals” as important processes during the formation of nephrite. Subsequent work described similar processes, suggesting that nephrite forms as “recrystallization products of thermal and cataclastic metamorphism”^24^, by metasomatism aided by tectonic movement^23^ or “metamorphism accompanying shearing”^22^. Leaming (1978) summarised the conditions required for nephrite formation as “a dynamic environment best produced by tectonic activity associated with faulting” combined with “changing pressure [and] temperature”^6^. Electron microscope studies of nephrite show that crystals are elongate and possess a range of shape- and crystallographic-preferred orientations, and that textures can include tightly-packed bundles of amphibole that are seemingly randomly oriented with respect to one another^2,31^. However, our understanding of the specific deformation processes leading to the formation of nephrite is limited, in part due to the difficulty of studying the microstructure of very fine-grained monomineralic aggregates, which has resulted in several competing hypotheses regarding the dominant grain-scale mechanisms at work^8–10,19,20,23,24,32,33^.

In this paper, we present a conceptual model for the evolution of nephrite jade that distinguishes four nephrite types based on mode of formation and textural characteristics. The model is derived from optical, scanning electron microscope (SEM), and electron backscatter diffraction (EBSD) analysis of 50 samples (sample catalogue in Supplementary Item 1 and Table S1), including six representative samples that are described in detail below (Table 1). Recognition of common microstructural characteristics in these samples, together with published descriptions of nephrite microstructure, leads us to suggest that many examples of massive nephrite may evolve from tremolite-actinolite vein networks due to the progressive development (and disruption) of metamorphic fabrics mediated by the activity of dissolution–precipitation.

**Table 1:** Summary of the six nephrite samples presented in detail in this study. Other samples are listed in Supplementary Item 2 and Table S1.

Sample	Location (latitude, longitude in WGS84)		Host serpentinite body	Structural setting	Sample mineralogy	Key references
MR16 primary vein nephrite	Mt Raddle, Westland, New Zealand	(− 44.241, 168.476)	Livingstone Fault, Dun Mountain Ophiolite (DMO)	Reaction zone between serpentinite shear zone and quartzofeldspathic schist	Tremolite, lizardite, chrysotile, Minor: magnetite, Cr-spinel	Refs^11,34–36^
JC13 folded vein nephrite	Jade Cove, California, USA	(35.928, − 121.469)	Franciscan Complex serpentinite	Reaction zone at contact of serpentinite blocks in metagraywacke mélange	Tremolite, lizardite, chrysotile, Minor: magnetite, Cr-spinel	Refs^10,37,38^
OU46117 crenulated nephrite	Whitcombe River, New Zealand	(− 43.067, 171.033)	Pounamu Ultramafics	Reaction zone at contact of lenses of serpentinite in a greenschist mélange	Tremolite	Refs^39–42^
OU65872 crenulated nephrite	Muddy Creek, Northwest Otago, New Zealand	(− 44.168, 169.280)	Ultramafic block in Haast quartzofeldspathic schist	Faulted contact between serpentinite and gabbro lens and quartzofeldspathic schist	Tremolite, minor: fuchsite, clinochlore	Refs^9,25^
TW04 foliated semi-nephrite	Hualien County, Taiwan	(23.862, 121.454)	Mafic to ultramafic tectonic blocks in the Yuli belt	Serpentinite shear zone	Tremolite	Refs^43^
HCF2 domainal/nodular nephrite	Hacket Creek, Nelson, New Zealand	(− 41.402, 173.239)	Serpentinised ultramafic portion of Dun Mountain Ophiolite (DMO)	Reaction zone between metagabbro and serpentinite in tectonised portion of DMO	Tremolite, Minor: Cr-spinel	

## Results

Below, we describe the microstructural characteristics of six representative samples, and then discuss possible evolutionary pathways that may account for the development of different types of nephrite. Table 1 includes details of the location, structural setting, and mineralogy of these six samples, and additional petrological and textural descriptions are provided in Supplementary Item 2.

### Type 1: Vein nephrite

#### Type 1a: primary vein nephrite

**Figure 1:**
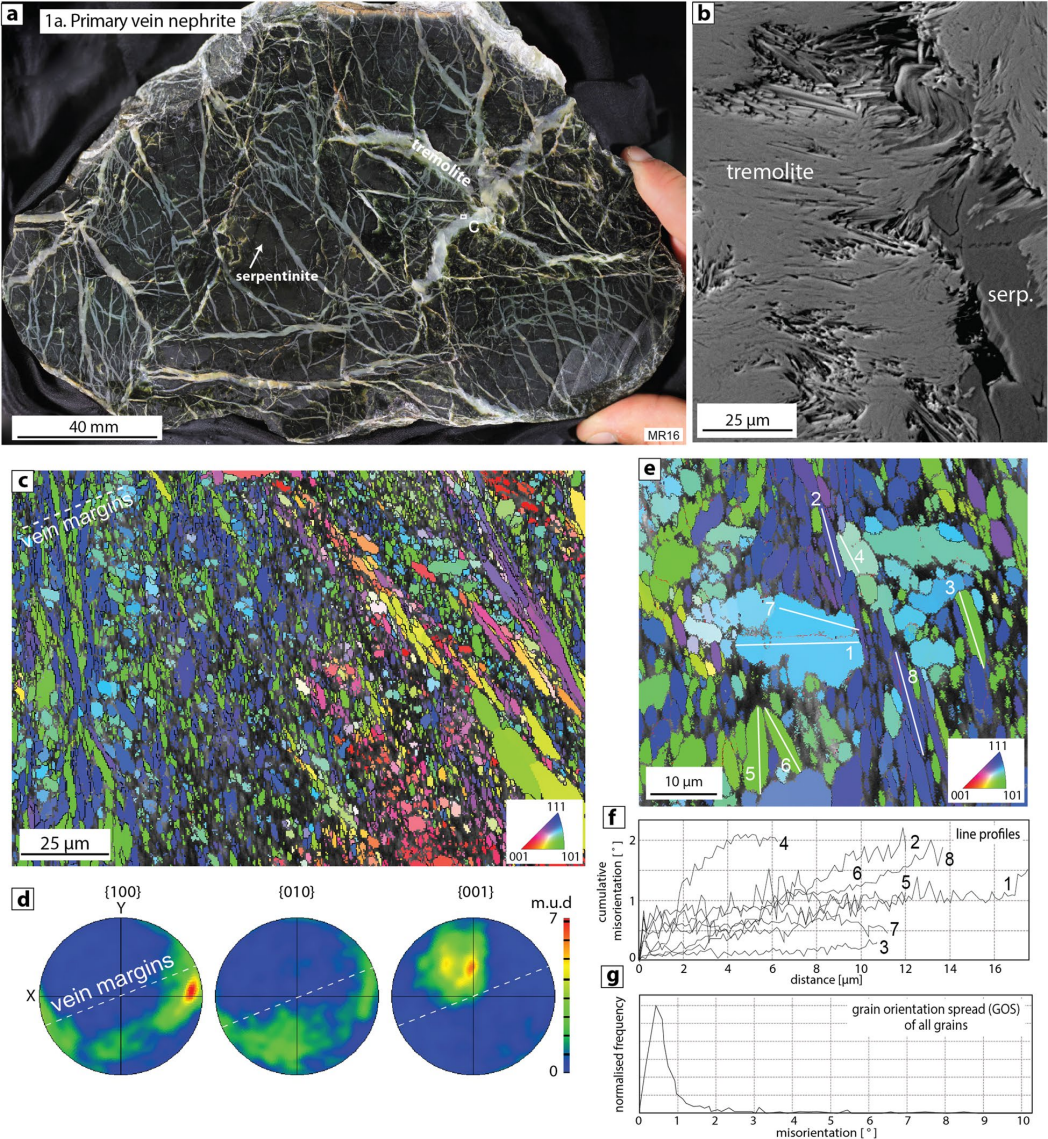
Type 1a primary vein nephrite (sample MR16). (**a**) Polished sample with sharp-walled, multi-generational tremolite veins cross-cutting massive serpentinite host rock, (**b**) SEM backscatter image (BS) of fibrous to acicular vein tremolite with inclusion of serpentinite, (**c**) EBSD map showing elongate to fibrous tremolite crystals lying sub-perpendicular to vein margins. In this and all subsequent EBSD maps, the pixels are coloured according to the inverse pole figure scheme (inset in bottom right) that shows which crystal direction lies parallel to the x-direction of the reference frame (i.e. horizontal in all images and pole figures). Red colours represent c-axis (001) parallel to x, green is 101 axis parallel to x, and blue is 111 axis parallel to x. Boundaries with > 10° misorientation are shown in black (e.g. grain boundaries) and boundaries with 5–10° of misorientation are shown in red (e.g. subgrain boundaries), (**d**) Pole figures showing contours of one point per grain from the EBSD map shown in part c. m.u.d = multiples of uniform distribution, (**e**) Detail of EBSD map showing the locations of line profiles used to measure internal misorientation within 8 tremolite crystals, (**f**) Plot of cumulative misorientation versus distance through individual tremolite crystals, (**g**) Internal misorientation (= grain orientation spread, GOS) of each grain shown in the EBSD map in part c. Very few grains have internal misorientation > 1°.

Tremolite formed by metasomatic reactions in serpentinite-bearing shear zones can crystallise via two main mechanisms:

1. Firstly, by direct crystallisation in veins (e.g., sample MR16; Fig. 1) that are often formed by cyclic hydrofracture^11,44^. Metasomatic reactions occurring between serpentinite and calcium- and silica-rich lithologies in serpentinite-bearing shear zones^11^, or between serpentinite blocks in a quartzofeldspatic mélange^39^, release fluid leading to repeated hydrofracturing and the formation of multi-generational vein networks^11,44^. Cross-cutting relationships indicate that vein networks often continue to form even during later stages of nephrite development. The veins are sharp-walled, monomineralic, cross-cutting (Fig. 1a), and contain elongate to fibrous acicular tremolite crystals that typically grow sub-perpendicular to vein margins in a lizardite- and chrysotile- dominated serpentinite host rock (Fig. 1b–d; Tarling et al.^11^). In the samples studied here, the tremolite crystals in nephrite veins are typically between 1 and 30 µm long with a width of between 0.5 and 5 µm (Fig. 1b–e). Tremolite c-axes (001) are clustered at large angles to vein margins, but show substantial spread due to “fanning” of tremolite aggregates (Fig. 1d). The a- (100) and b-axes (010) are rotated around the c-axes resulting in girdle patterns (Fig. 1d). Line profiles of cumulative misorientation made through individual tremolite crystals show negligible to very low degrees of internal misorientation and a lack of low-angle or subgrain boundaries within crystals (< 2°; Fig. 1e,f). The overall frequency distribution of misorientation within all measured grains shows that a large majority of grains have < 1° of internal misorientation (Fig. 1g).
2. Secondly, by dispersed growth of tremolite due to replacement of pre-existing phases, including serpentine minerals (lizardite, chrysotile) in the serpentinite matrix (e.g., sample JC13; Fig. S1) or other minerals found in wall rocks or exotic blocks contained in serpentinite shear zones^10^. Crystal sizes are broadly similar to those found in the vein tremolite described above.

These two tremolite crystallisation and growth mechanisms commonly operate concurrently, which can lead to the complete replacement of the serpentinite matrix by aggregates of acicular tremolite, accompanied by development of tremolite vein networks.

#### Type 1b: folded vein nephrite with incipient crenulation cleavage

**Figure 2:**
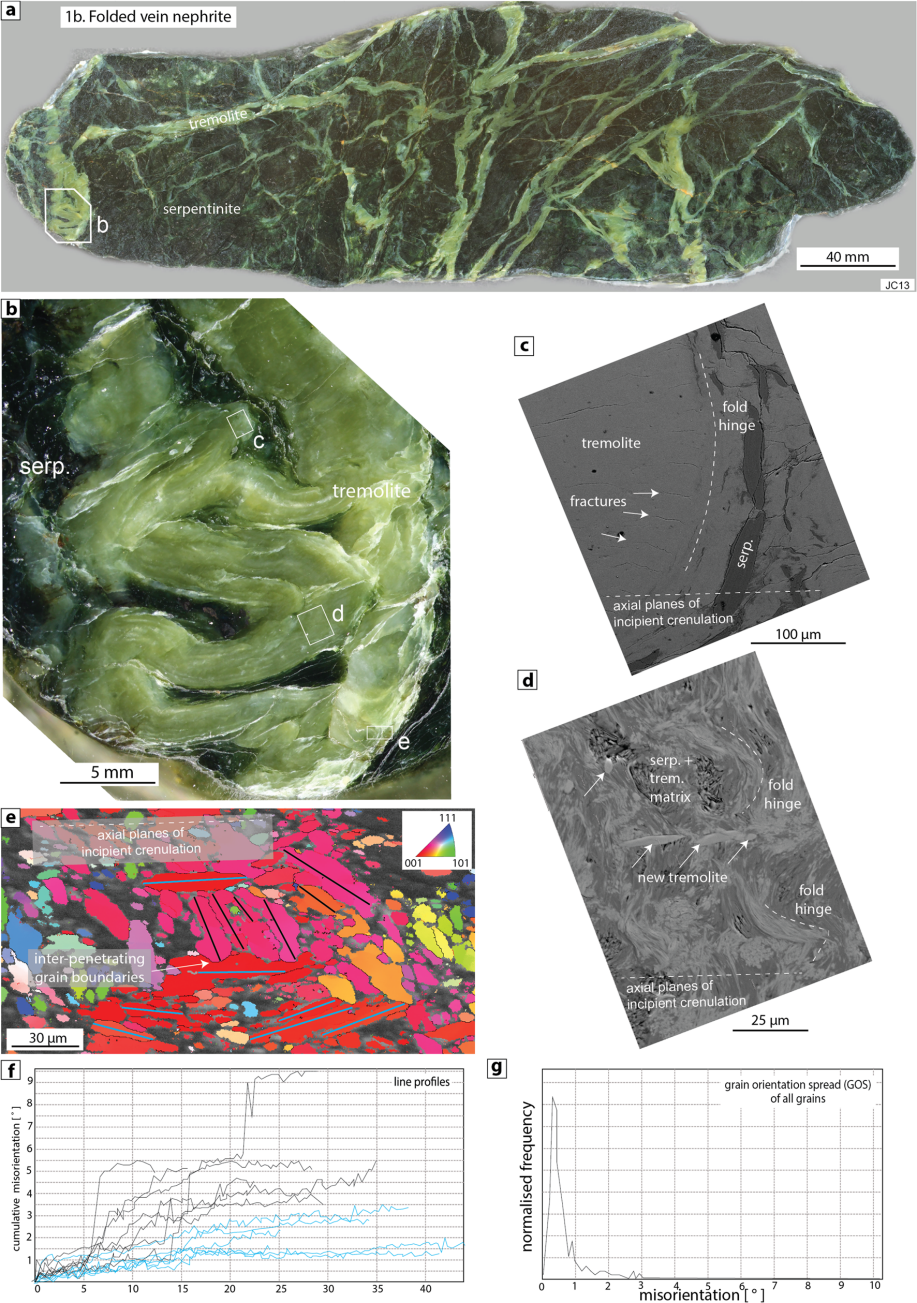
Type 1b folded vein nephrite (sample JC13). (**a**,**b**) Polished sample showing a folded tremolite vein containing a range of textures that define an incipient axial planar cleavage (sub-horizontal in all images), (**c**) SEM backscatter image of brittle fractures (white arrows) developed in the outer arc of a nephrite fold hinge. The fractures are sub-parallel to fold axial planes, (**d**) SEM backscatter image of folded serpentine-tremolite matrix. New euhedral tremolite crystals (white arrows) grew sub-parallel to fold axial planes and incipient crenulation, (**e**) EBSD map of fold hinge region coloured according to the inverse pole figure shown in top right. Tremolite crystals at high angles to one another meet along inter-penetrating grain boundaries (white arrow). Black and blue lines show the locations of line profiles used to measure internal misorientation within 13 tremolite crystals, (**f**) Profiles of cumulative internal misorientation through the grains shown in part e. Newly grown tremolite crystals sub-parallel to fold axial planes (blue lines) are relatively internally strain-free compared to older matrix tremolite that experienced folding (black line profiles), (**g**) Internal misorientation (= grain orientation spread, GOS) of each grain shown in the EBSD map in part e.

**Figure 3:**
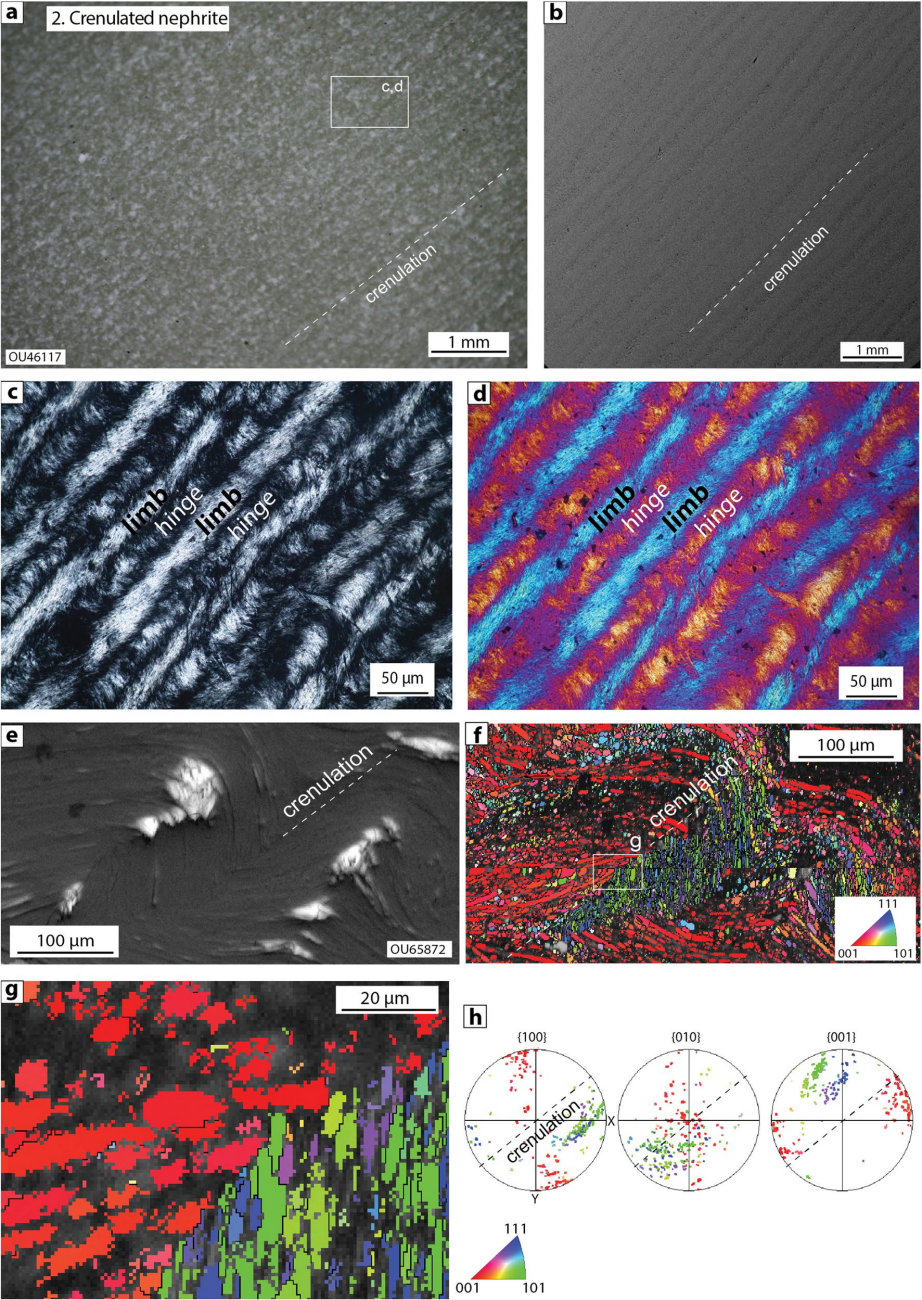
Type 2 crenulated nephrite (samples OU46117 (parts a-d) and OU65872 (parts e–h)),(**a**) Polished sample of crenulated nephrite with faint crenulation cleavage, (**b**) SEM backscatter image showing crenulation defined by a weak striping, which reflects the variable polish on fold hinges and limbs, (**c**,**d**) Thin section images of regularly spaced crenulations defined by alternating fold limbs and hinges in crossed-polarised light (**c**) and with the gypsum plate inserted (**d**), (**e**) SEM secondary-electron image of chevron or semi-chevron fold hinge regions, (**f**) EBSD map of same region shown in part e coloured according to the inverse pole figure (bottom right), (**g**) Detail of fold hinge showing sharp transition in both shape- and crystallographic-preferred orientation across chevron fold hinge, (**h**) Pole figure data from map in (**g**) highlighting the different crystallographic-preferred orientations in the two different fold limbs.

**Figure 4:**
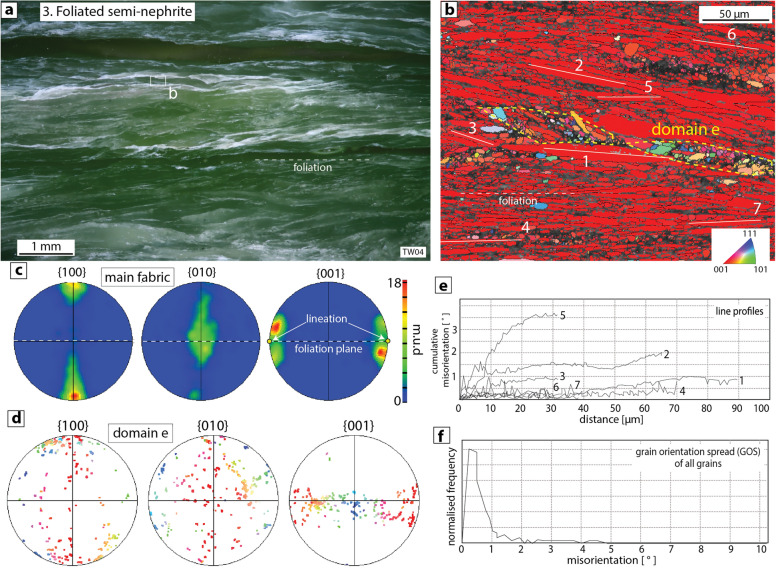
Type 3 foliated semi-nephrite (sample TW04), (**a**) Polished sample of foliated semi-nephrite, (**b**) EBSD map showing dominance of strongly aligned tremolite crystals (red on map) that comprise the main schistosity. Also present are elongate domains (e.g. domain e) that contain tremolite crystals with a wider range of shape and crystallographic orientations than displayed by the main fabric, (**c**) Contoured pole figures (one point per grain) showing data from the main fabric. Tremolite laths and needles have their c-axes (001) strongly aligned parallel to the foliation and lineation, (**d**) Pole figures showing all data from domain e, (**e**) Line profiles of cumulative misorientation through 7 tremolite crystals shown in part b, (**f**) Internal misorientation (= grain orientation spread, GOS) of each grain shown in the EBSD map in part b.

Folding during deformation in shear zones leads to the formation of an incipient crenulation cleavage defined by: (1) alignment of the limbs and axial planes of (sometimes ptygmatic) folds that develop from pre-existing primary nephrite veins (e.g. sample JC13; Fig. 2a–b), (2) brittle fractures that form most commonly around the outer arc of folded veins, and lie sub-parallel to the axial planes of folds (Fig. 2c), (3) folding of the tremolite-serpentinite matrix surrounding veins (Fig. 2d), and (4) growth of new tremolite crystals sub-parallel to the axial planar fabric (Fig. 2d; Supplementary Item 3, Fig. S1). New crystals are often euhedral, crosscut the surrounding matrix (Fig. 2d, Supplementary Item 3, Fig. S1), and have distinct chemical compositions from the matrix tremolite (Supplementary Item 3). ESBD analysis shows that tremolite crystals in fold hinge regions meet along inter-penetrating grain boundaries between individual crystals, especially if the crystals are at high angles to one another (Fig. 2e). Additionally, newly-grown tremolite crystals that lie sub-parallel to the incipient axial planar fabric have smaller degrees of internal misorientation (< 3°) than older crystals that were deformed during the folding process (up to 9°; Fig. 2e–g).

### Type 2: crenulated nephrite

In cases where deformation combined with pervasive metasomatic reactions leads to the formation of massive nephrite layers or lenses^1,6^, at least three texturally distinct varieties of nephrite (types 2–4 here) can be recognised based on our own observations and previous descriptions of nephrite texture. Some nephrites contain a well-organised spaced crenulation cleavage (Fig. 3) defined by cleavage domains that represent fold limbs composed of strongly aligned tremolite crystals, separated by microlithons that represent preserved fold hinges (e.g. OU46117 and OU65872, Fig. 3; refs^9,40^ ). In one example shown here (Fig. 3a–d) there is a smooth and progressive change in the orientation of tremolite crystals across fold hinges and limbs, and each crenulation is on the order of 50 µm wide. In the other example, the folds have a chevron geometry and there is a sharp transition in both shape and crystallographic preferred orientation of tremolite crystals across the fold hinges (Fig. 3e–h). In both of these samples, tremolite crystals are ~ 1–50 µm long, 1–10 µm wide, contain negligible to very low degrees of internal misorientation (< 2°), and lack any subgrains (Fig. 3f,g).

### Type 3: foliated semi-nephrite/tremolite schist

In strongly foliated semi-nephrites, which are fine-grained tremolite rocks whose grains lack the interlocking characteristic of nephrites and are texturally equivalent to many fine-grained “tremolite schists”^37^, the dominant schistosity is defined by well-aligned laths and needles of tremolite (e.g. TW04; Fig. 4a,b), which in some cases also define a macroscopic lineation. The crystallographic preferred orientation (CPO) of the main schistosity is characterised by a strong alignment of the c-axes (001) parallel to the bulk foliation plane and, if present, the lineation (Fig. 4b,c). The a- (100) and b- (010) axes define weak girdles orthogonal to the c-axes, with well-defined maxima perpendicular (a-) and parallel (b-) to the foliation plane, respectively (Fig. 4c). However, although the main schistosity is dominant, there are also domains up to 50 µm wide that contain tremolite crystals with a wider range of shape and crystallographic orientations than displayed by the main fabric (e.g. domain e in Fig. 4b,d). Crystals throughout generally show negligible to very minor degrees of internal misorientation (Fig. 4e,f), with no significant development of low-angle boundaries or subgrains (Fig. 4b).

### Type 4: nodular or domainal nephrite

**Figure 5:**
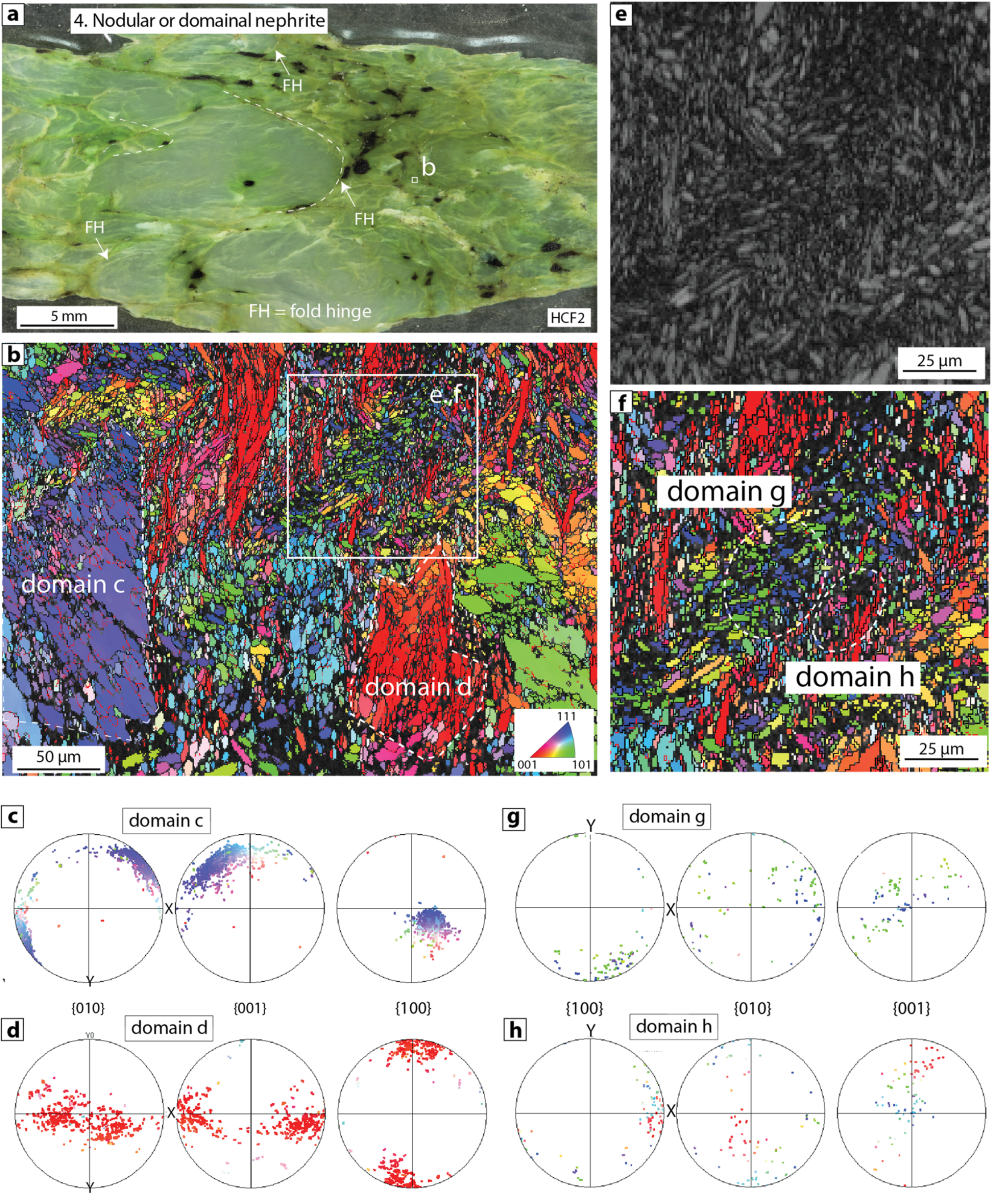
Type 4 domainal/nodular nephrite (sample HCF2). (**a**) Polished sample of nodular/domainal nephrite. The left-hand side of the sample contains several macroscopic fold hinges (FH) defining a nodular texture, whereas the right-hand side of the sample contains domainal textures, (**b**) EBSD map showing two distinct domains (**c** and **d**) with angular boundaries comprising tremolite aggregates with well-defined shape- and crystallographic-preferred orientations. The tremolite matrix between domains preserves a crenulated fabric, (**c**,**d**) Pole figures from domains c and d highlighting the marked difference in crystallographic orientations of the constituent amphibole aggregates, (**e**) EBSD band contrast map and (**f**) corresponding EBSD map of a crenulated region of the matrix, (**g**,**h**) Pole figures from domains g and h within the preserved crenulated fabric, highlighting the different crystal orientations in two adjacent fold limbs.

Nodular or domainal nephrite has the most complex range of microstructures (e.g. HCF2; Fig. 5a). Samples consist entirely of tremolite aggregates that are arranged into distinct microstructural domains with a range of shapes and sizes, surrounded by a fine-grained matrix of acicular tremolite (Fig. 5a,b). In some samples, domains have the shape of relict fold hinges which impart a nodular texture to the nephrite (FH in Fig. 5a). In other cases, the boundaries between domains and the surrounding matrix define angular clasts (e.g. domains c and d in Fig. 5b). Internally, individual domains comprise aggregates of fine-grained acicular to elongate tremolite with well-defined shape- and crystallographic-preferred orientations (Fig. 5b–d). However, the SPO and CPO in different domains are rotated with respect to each other, and also have different orientations compared to the surrounding tremolite matrix (Fig. 5b–d). The matrix between domains can preserve areas with a crenulated or folded fabric (Fig. 5e–h), but in this case the crenulation is not as pervasive and consistent as in the Type 2 nephrites described above.

## Discussion

**Figure 6:**
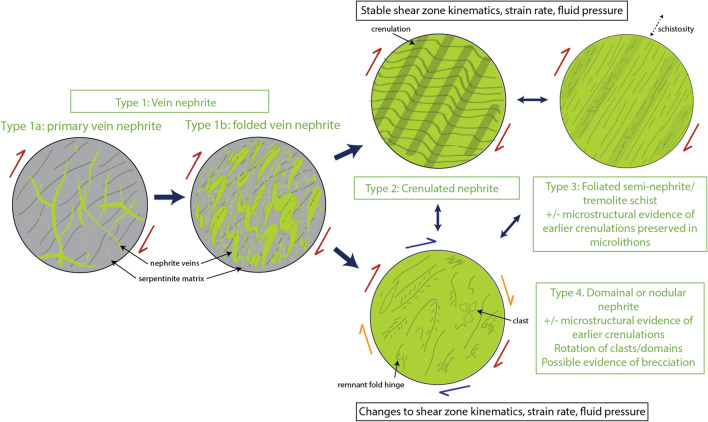
Conceptual evolutionary model and proposed classification of serpentinite-derived nephrite jade based on mode of formation and microstructural characteristics.

We interpret the texture of our analysed nephrite samples to represent snapshots of a progressive textural evolution similar to that documented in other deformed and fine-grained metamorphic rocks that develop crenulated and foliated fabrics under fluid-present, greenschist-facies conditions (Fig. 6; refs^45–47^). We suggest that nephrites can form:

1. By direct precipitation in veins (as Type 1a *primary vein nephrites*),
2. Due to the development and evolution of metamorphic fabrics during folding and deformation in shear zones (Types 1b, 2, 3), with dissolution–precipitation as the key process mediating fabric evolution, or,
3. By brecciation and disruption of previously-formed nephrites containing metamorphic fabrics (type 4; Fig. 6).

In the following three paragraphs, we discuss each of these scenarios separately.

The common spatiotemporal association between massive nephrite layers/lenses and tremolite-actinolite vein networks suggests that many nephrites may (at least partly) originate from veins^6,8,13,26–29,32,39,43,48–51^. Rapid tremolite precipitation in a dynamic shear zone environment favours growth of µm-sized laths and needles, perhaps indicating unconstrained radiating growth^52^. Several examples of nephrite in the literature contain crack-seal banding and slickenfibre-coated surfaces suggesting that they represent fragments of primary (Type 1a *primary vein nephrite*) or folded tremolite veins (Type 1b *folded vein nephrite*; Fig. 6; e.g. refs^8,26–28,32,39,43^ ). However, this does not preclude the possibility that nephrite types 2–4 may also develop without pre-existing veins, either from disseminated tremolite that grows at the expense of serpentine (or other phases), or from tremolite-bearing reaction rinds that are subsequently deformed.

In our conceptual model, one pathway leading to the development of massive nephrite layers/lenses occurs if shear zone kinematics and other conditions (e.g. strain rate) are stable for a period of time, during which progressively folded veins and metasomatic reaction zones evolve towards a spaced crenulation cleavage (Type 2 *crenulated nephrite*), which may ultimately be consumed by a new penetrative planar fabric containing remnant microstructures preserved in microlithons (Type 3 *foliated semi-nephrite/tremolite schist*). The tremolite crystals in types 1–3 nephrite samples: (1) are often elongate to fibrous acicular crystals up to tens of microns long and a few microns wide; (2) contain negligible to very low degrees of internal misorientation, typically ≤ 2°; (3) lack significant subgrain development or other indicators of recovery and recrystallisation such as gradational internal misorientation^53^; and (4) have grain boundaries that are often interlocking and, in some instances, interpenetrating. Additionally, folded and crenulated samples contain evidence for the growth of chemically distinct and relatively strain-free tremolite crystals that contribute to the development of an incipient axial planar fabric (Supplementary Item 3, Fig. S1, Table S2), while strongly foliated semi-nephrites contain tremolite with well-defined shape and crystallographic preferred orientations that define the main schistosity and lineation. Collectively, we interpret these observations to indicate that dissolution–precipitation is the most important long-term deformation mechanism during the formation of nephrite types 1b, 2 and 3. However, it is also evident that dissolution–precipitation is sometimes accompanied by brittle fracturing that occurs within the hinge regions of folded nephrite layers (e.g. type 1b folded nephrite vein in Fig. 2c). Our interpretation of dissolution–precipitation as the dominant deformation process during the evolution of nephrite fabrics is consistent with previous studies of deformed amphibole-bearing rocks, which report a tendency for amphibole to preferentially deform by dissolution–precipitation at temperatures of < 650 °C, sometimes accompanied by brittle fracture (e.g., refs^54–58^ ).

A second and perhaps more common pathway to develop massive nephrite occurs if shear zone kinematics change, or strain rate or fluid pressure transients occur, resulting in disruption or brecciation of a previously-formed folded, crenulated, or schistose fabric (Fig. 6). In addition to the sample described here, brecciated nephrites are reported from other localities in New Zealand^8,9^ and the Apennine Mountains in Italy^59^ and consist of angular clasts of nephrite surrounded by a low-porosity nephritic matrix, similar to the area shown in Fig. 5b. Individual microstructural domains in these samples have strong shape- and crystallographic-preferred orientations, which we interpret to have formed mainly by a dissolution–precipitation process as described above for nephrite types 1b, 2 and 3. However, evidence for angular domain boundaries and disorganised arrangement of shape- and crystallographic-orientations within individual domains suggests that the previously-formed metamorphic fabrics suffered disruption due to brittle fracturing and clast rotation (e.g. cataclasis, brecciation). Disruption and brecciation results in Type 4 *domainal or nodular nephrites* (Fig. 6), although microstructural evidence for previously-formed fabrics can be preserved as remnant fold hinges, partially-preserved crenulation cleavages, or discrete microstructural domains with strong SPO and CPO. Although disruption and brecciation of previously-formed fabrics is the key process involved in producing type 4 nephrites, the presence of a pure nephrite matrix in between clasts also suggests that precipitation of tremolite must rapidly seal any pore space developed by fracturing and brecciation, resulting in a low-porosity, fine-grained, well-cemented and monomineralic rock.

Because nephrite is formed exclusively within or close to the margins of shear zones^1,6–10,22^, it is likely that many nephrites experience numerous cycles of fabric formation and destruction, which could be accompanied at any stage by the formation of new vein networks. Nephrites that form in large-displacement shear zones are thus likely to experience multiple cycles of fabric evolution, which may result in complicated microstructures. The model framework presented here is broadly consistent with previous hypotheses for the formation of nephrite, which have invoked the concurrent role of metasomatism and deformation^1,8–10,19–30^, and identified key shear zone processes such as dissolution^20^ and brecciation^9^. However, our model builds on these previous observations by specifically identifying veining and dissolution–precipitation as playing a dominant role in the formation of many nephrites, and by proposing a conceptual framework for the progressive textural evolution of nephrite that is based on well-established metamorphic concepts (Fig. 6). This model will not provide a satisfactory explanation for the textures observed in all types of nephrite. For example, due to the challenges of performing SEM and EBSD-based analyses on very fine-grained monomineralic samples, our sample set under-represents the highest grades of nephrite which appear to consist of ultra-fine grained tremolite aggregates and an overall near-homogenous texture at the hand specimen and thin section scale^1,6,31^. Future work could involve further micro- and nano-structural analysis combined with mechanical testing of nephrites, which will help to determine how fabric and crystallography relate to material properties such as fracture toughness.

## Methods

### Sample preparation

Most samples were obtained as thin sections from existing collections. New samples were cut perpendicular to the foliation and parallel to the lineation (where present) and prepared either as thin sections ground to 30 µm thickness, or as briquettes with nephrites embedded in epoxy resin. In cases where no foliation or lineation was perceptible, multiple thin sections and briquettes were prepared from a range of orientations to ensure that a bias was not introduced from selecting a plane in which a lower degree of grain orientations is present (e.g., a cut coinciding with a foliation plane). Thin sections and briquettes were polished progressively with diamond paste, up to a 0.3 µm polish and finally polished with a colloidal silica in a reactive alkaline suspension (OP-S, 0.25 µm, Struer).

### Microstructural observations, EBSD, and EDS analysis

Microstructural observations were initially made using a standard research-grade polarised optical microscope. Selected samples were analysed using a Zeiss Sigma Field-Emission Gun scanning electron microscope in the Otago Micro and Nanoscale Imaging (OMNI) facility at the University of Otago. Backscatter electron (BE) and secondary electron (SE) images were acquired using a 15 keV accelerating voltage and 6.6–8 mm working distance. Electron Backscatter Diffraction (EBSD) data were acquired using an HKL Synergy Integrated EDS/EBSD system (Oxford Instruments) with an accelerating voltage of 30 kV and an aperture of 300 μm. Post-acquisition EBSD data processing was performed in Channel 5 software (Oxford Instruments) and the MTEX toolbox for MATLAB. Energy-Dispersive X-Ray Spectroscopy (EDS) measurements of chemical composition were acquired with an acceleration voltage of 15 kV, a beam current of approximately 1 nA, a live count time of 60 s, and a working distance of 8.5 mm.

## Supplementary Information

Supplementary Information.

## References

[1] Harlow GE, Sorensen SS (2005). Jade (nephrite and jadeitite) and serpentinite: Metasomatic connections. Int. Geol. Rev..

[2] Bradt RC, Newnham RE, Biggers JV (1973). The toughness of jade. Am. Mineral. J. Earth Planet. Mater..

[3] Rowcliffe DJ, Frühauf V (1977). The fracture of jade. J. Mater. Sci..

[4] Wu CC, McKinney KR, Rice RW (1990). Strength and toughness of jade and related natural fibrous materials. J. Mater. Sci..

[5] Wilkins CJ, Tennant WC, Williamson BE, McCammon CA (2003). Spectroscopic and related evidence on the coloring and constitution of New Zealand jade. Am. Mineral..

[6] Leaming, S. F. *Jade in Canada*. (Geological Survey of Canada Paper v. 78–19. Energy, Mines and Resources Canada, 1978).

[7] Coleman RG (1967). Low-temperature reaction zones and alpine ultramfic rocks of California oregon and Washington. Geol. Surv. Bull..

[8] Coleman RG (1966). New Zealand serpentinite and associated metasomatic rocks: Bulletin. New Zeal. Geol. Surv..

[9] Cooper AF (1995). Nephrite and metagabbro in the haast schist at muddy creek, northwest Otago, New Zealand. New Zeal. J. Geol. Geophys..

[10] Crippen RA (1951). Nephrite jade and associated rocks of the Cape San Martin region, Monterey County, California. Calif. Div. Mines Geol. Spec. Publ. Spec. Rep..

[11] Tarling MS, Smith SAF, Scott JM (2019). Fluid overpressure from chemical reactions in serpentinite within the source region of deep episodic tremor. Nat. Geosci..

[12] Read HH (1934). On zoned associations of antigorite, talc, actinolite, chlorite, and biotite in Unst, Shetland Islands. Mineral. Mag. J. Mineral. Soc..

[13] Chidester AH (1962). Petrology and geochemistry of selected talc-bearing ultramafic rocks and adjacent country rocks in north-central Vermont. U.S. Geol. Surv. Prof. Pap..

[14] Curtis CD, Brown PE (1971). Trace element behavior in the zoned metasomatic bodies of Unst Shetland. Contrib. Mineral. Petrol..

[15] Curtis CD, Brown PE (1969). The metasomatic development of zoned ultrabasic bodies in Unst Shetland. Contrib. to Mineral. Petrol..

[16] Matthews DW (1967). Zoned ultrabasic bodies in the Lewisian of the Moine Nappe in Skye. Scott. J. Geol..

[17] Fowler MB (1981). The metasomatic development of zoned ultramafic balls from Fiskenaesset West Greenland. Mineral. Mag..

[18] Francis GH (1955). Zoned hydrothermal bodies in the serpentinite mass of glen Urquhart (Inverness-shire). Geol. Mag..

[19] Finlayson AM (1909). The nephrite and magnesian rocks of the South Island of New Zealand. Q. J. Geol. Soc..

[20] Turner FJ (1935). Geological investigations of the nephrites, serpentines, and related" green stones" used by the Maoris of Otago and South Canterbury. Trans. R. Soc. New Zeal..

[21] Clarke, F. W. & Merrill, G. P. On nephrite and jadeite. In *Proc. United States Natl. Museum* (1889).

[22] Adams CJ, Beck RJ, Campbell HJ (2007). Characterisation and origin of New Zealand nephrite jade using its strontium isotopic signature. Lithos.

[23] Bloomfield K (1958). The Chimwadzulu Hill ultrabasic body, southern Nyasaland. South Afr. J. Geol..

[24] Holland, S. S. Jade in British Columbia. *BC Minist. Energy, Mines Pet. Resour. Annu. Rep.* 119–126 (1961).

[25] Cooper AF (1976). Concentrically zoned ultramafic pods from the Haast schist zone, South Island, New Zealand. New Zeal. J. Geol. Geophys..

[26] Simandl GJ, Riveros CP, Schiarizza P (1999). Nephrite (jade) deposits, mount Ogden area, central British Columbia (NTS 093N 13W). Geol. Fieldwork.

[27] Prokhor SA (1991). The genesis of nephrite and emplacement of the nephrite-bearing ultramafic complexes of East Sayan. Int. Geol. Rev..

[28] Hockley JJ, Birch WD, Worner HK (1978). A nephrite deposit in the great serpentine belt of New South Wales. J. Geol. Soc. Aust..

[29] Kolesnik IN (1970). Nephrites of Siberia.

[30] Campbell, G. P., Miskelly, G. M., Coulson, S. A., Yaxley, G. M. & Curran, J. M. Elemental analysis of New Zealand Nephrite Jade (pounamu) for forensic purposes. (2008).

[31] Dorling M, Zussman J (1985). An investigation of nephrite jade by electron microscopy. Mineral. Mag..

[32] Chesterman, C. W. Nephrite in Marin County. *Calif. Div. Mines Geol. Spec. Publ. Spec. Rep. 10-B* (1951).

[33] Cooper, A. F. Origin and evolution of nephrites, diopsidites and giant diopside crystals from the contact zones of the Pounamu Ultramafics, Westland, New Zealand. *New Zeal. J. Geol. Geophys.*10.1080/00288306.2022.2050771 (2022).

[34] Tarling, M. S. *et al.* The internal structure and composition of a plate boundary-scale serpentinite shear zone: The livingstone fault, New Zealand. Solid Earth 10.5194/se-2019-62 (2019).

[35] Scott JM (2019). Element and Sr–O isotope redistribution across a plate boundary-scale crustal serpentinite mélange shear zone, and implications for the slab-mantle interface. Earth Planet. Sci. Lett..

[36] Tarling MS, Smith SAF, Viti C, Scott JM (2018). Dynamic earthquake rupture preserved in a creeping serpentinite shear zone. Nat. Commun..

[37] Hirauchi K, Yamamoto Y, den Hartog SAM, Niemeijer AR (2020). The role of metasomatic alteration on frictional properties of subduction thrusts: An example from a serpentinite body in the Franciscan complex California. Earth Planet. Sci. Lett..

[38] King RL, Kohn MJ, Eiler JM (2003). Constraints on the petrologic structure of the subduction zone slab-mantle interface from Franciscan complex exotic ultramafic blocks. Bull. Geol. Soc. Am..

[39] Cooper AF, Reay A (1983). Lithology, field relationships, and structure of the pounamu ultramafics from the whitcombe and hokitika rivers, Westland, New Zealand. New Zeal. J. Geol. Geophys..

[40] Popham, T. B. Pounamu : Characterisation and resource assessment; Scott Basin, Wakatipu, New Zealand. (Unpublished Thesis, University of Otago, New Zealand, 2006).

[41] Ireland TR, Reay A, Cooper AF (1984). The pounamu ultramafic belt in the diedrich range, Westland, New Zealand. New Zeal. J. Geol. Geophys..

[42] Koons PO (1981). A study of natural and experimental metasomatic assemblages in an ultramafic-quartzofeldspathic metasomatic system from the haast schist, South Island New Zealand. Contrib. Mineral. Petrol..

[43] Kuo, L.-W. Is the birth of Taiwan nephrite related to plate tectonics? in *Taiwan Jade: Past and Present* 100–119 (National Taiwan Museum, 2020).

[44] Nishiyama T, Shiosaki CY, Mori Y, Shigeno M (2017). Interplay of irreversible reactions and deformation: A case of hydrofracturing in the rodingite–serpentinite system. Prog. Earth Planet. Sci..

[45] Bell TH, Rubenach MJ (1983). Sequential porphyroblast growth and crenulation cleavage development during progressive deformation. Tectonophysics.

[46] Naus-Thijssen FMJ, Johnson SE, Koons PO (2010). Numerical modeling of crenulation cleavage development: A polymineralic approach. J. Struct. Geol..

[47] Mamtani MA, Karanth RV, Greiling RO (1999). Are crenulation cleavage zones mylonites on the microscale?. J. Struct. Geol..

[48] Kislov EV, Erokhin YV, Popov MP, Nikolayev AG (2021). Nephrite of bazhenovskoye chrysotile-asbestos deposit, middle urals: Localization mineral composition and color. Minerals.

[49] Burtseva MV, Ripp GS, Posokhov VF, Zyablitsev AY, Murzintseva AE (2015). The sources of fluids for the formation of nephritic rocks of the southern folded belt of the Siberian Craton. Dokl. Earth Sci..

[50] Lindley, I. D. & White, P. J. S. A review of the occurrence of and potential for jade in the New Guinea Mobile Belt. *Aust. J. Earth Sci.* 1–20 (2021).

[51] Gil G (2015). Origin of serpentinite-related nephrites from Gogołów-Jordanów Massif Poland. Geol. Q..

[52] Cluzel D (2020). Slab-derived origin of tremolite–antigorite veins in a supra-subduction ophiolite: The peridotite nappe (new caledonia) as a case study. Int. J. Earth Sci..

[53] Halfpenny, A., Prior, D. J. & Wheeler, J. Using electron backscatter diffraction (EBSD) to measure misorientation between ‘parent’and ‘daughter’grains. Implications for recrystallisation and nucleation. in *Materials Science Forum* vol. 467 573–578 (Trans Tech Publ, 2004).

[54] Berger A, Stünitz H (1996). Deformation mechanisms and reaction of hornblende: Examples from the Bergell tonalite (central Alps). Tectonophysics.

[55] Imon R, Okudaira T, Fujimoto A (2002). Dissolution and precipitation processes in deformed amphibolites: An example from the ductile shear zone of the Ryoke metamorphic belt SW Japan. J. Metamorph. Geol..

[56] Giuntoli F, Menegon L, Warren CJ (2018). Replacement reactions and deformation by dissolution and precipitation processes in amphibolites. J. Metamorph. Geol..

[57] Lee, A. L., Stünitz, H., Soret, M. & Battisti, M. A. Dissolution precipitation creep as a process for the strain localisation in mafic rocks. *J. Struct. Geol.* 104505 (2022).

[58] Imon R, Okudaira T, Kanagawa K (2004). Development of shape-and lattice-preferred orientations of amphibole grains during initial cataclastic deformation and subsequent deformation by dissolution–precipitation creep in amphibolites from the Ryoke metamorphic belt SW Japan. J. Struct. Geol..

[59] Kalkowsky E (1906). Der Nephrit des Bodensees.

