# Supplementary material for: An evolutionary model and classification scheme for nephrite jade based on veining, fabric development, and the role of dissolution–precipitation
Source: Sci Rep. 2022 May 12;12:7823. doi: 10.1038/s41598-022-11560-7 (PMC9098473; doi:10.1038/s41598-022-11560-7)
Supplement: Supplementary file 1 — Supplementary Information. [file 41598_2022_11560_MOESM1_ESM.docx]

Supplementary Materials for

**An evolutionary model and classification scheme for nephrite jade based on veining, fabric development, and the role of dissolution-precipitation**

**Matthew S. Tarling^1,2^, Steven A.F. Smith^1^, Marianne Negrini^1^, Li-Wei Kuo^3^, Wei-Hsin Wu^3^, Alan F. Cooper^1^**

*^1^* *Department of Geology, University of Otago, Dunedin, New Zealand*

*^2^Department of Earth and Planetary Sciences, McGill University, Montréal, Québec, Canada*

*^3^ Department of Earth Sciences, National Central University, Taoyuan, Taiwan*

**Supplementary Item 1: Nephrite sample catalogue**

Table S1 contains a list of samples that were examined in this study. Sample identifiers with the prefix OU belong to the University of Otago Geology Department collection. Samples which also have corresponding material lodged in the Otago Museum collection are noted. The first six samples identified in **bold** are featured in the main figures and described in the main manuscript.

Nephrite is part of the family of rocks known as pounamu and is a taonga in Māori culture. Under the Ngāi Tahu Act of 1997 (the Pounamu Vesting Act), ownership of all pounamu in the Takiwa of Ngāi Tahu and the adjacent territorial seas has been returned to Ngāi Tahu. Samples of pounamu included in the sample set used in this study are part of the Geology Department collection and were collected prior to the Ngāi Tahu Act of 1997. Sample identifiers noted with an asterisk (*) represent likely Māori artifacts (based on archival notes), small flakes, partially worked or discarded pounamu which were subsampled during the archival process in the early 20^th^ Century.

Table S1: Catalogue of samples examined in this study.

| ***Sample number*** | ***Collection(s)*** | ***Collector*** | ***Location*** | ***Year collected*** | ***Nephrite type*** | ***Reference publication*** |
| --- | --- | --- | --- | --- | --- | --- |
| **MR16** | - | M.S. Tarling | Mount Raddle, NZ | 2016 | primary vein nephrite | Tarling et al. 2019 |
| **JC13** | - | M.S. Tarling & M. Demurtas | Jade Cove, California, USA | 2017 | folded vein nephrite | Crippen, 1951 |
| **OU46117** | University of Otago | A.F. Cooper | Whitcombe River, NZ | 1976 | crenulated nephrite | Cooper & Reay, 1983 |
| **OU65872** | University of Otago | A.F. Cooper | Muddy Creek, NW Otago, NZ | 1988 | crenulated nephrite | Cooper 1995 |
| **TW04** | - | LW Kuo | Hualien County, Taiwan | 2018 | Foliated semi-nephrite/tremolite schist | Kuo, 2020 |
| **HCF2** | - | M.S. Tarling | Hacket Creek float, Nelson, NZ | 2018 | Domainal or nodular nephrite | - |
| JC01 | - | M.S. Tarling & M. Demurtas | Jade Cove, California, USA | 2017 | vein nephrite | - |
| JC03 | - | M.S. Tarling & M. Demurtas | Jade Cove, California, USA | 2017 | Domainal or nodular nephrite | - |
| JC13 | - | M.S. Tarling & M. Demurtas | Jade Cove, California, USA | 2017 | vein nephrite | - |
| RS22 | - | M.S. Tarling | Red Spur, Westland, NZ | 2017 | vein nephrite | Tarling et al., 2019 |
| TW01 | - | LW Kuo | Hualien County, Taiwan | 2018 | Foliated semi-nephrite/tremolite schist | Kuo, 2020 |
| TW02 | - | LW Kuo | Hualien County, Taiwan | 2018 | Foliated semi-nephrite/tremolite schist | Kuo, 2020 |
| TW03 | - | LW Kuo | Hualien County, Taiwan | 2018 | Foliated semi-nephrite/tremolite schist | Kuo, 2020 |
| OU1364 | University of Otago | unknown | Upper Ford, Jackson River, NZ | pre-1935 | Foliated semi-nephrite/tremolite schist | Turner, 1935 |
| OU1370 | University of Otago | unknown | Upper Ford, Jackson River, NZ | pre-1935 | Foliated semi-nephrite/tremolite schist | Turner 1933, 1935 |
| OU1379 | University of Otago | unknown | Martyr Bridge & Jackson Saddle, NZ | pre-1935 | Foliated semi-nephrite/tremolite schist | Turner, 1935 |
| OU1442 | University of Otago | unknown | Routeburn, NW Otago, NZ | pre-1935 | Domainal or nodular nephrite | Turner, 1935 |
| OU1800* | University of Otago / Otago Museum (D 33. 1382) | unknown | Otago Beaches, NZ | pre-1935 | Domainal or nodular nephrite | Turner, 1935 |
| OU1801* | University of Otago / Otago Museum (D 30. 1746) | unknown | Milford, South Canterbury, NZ | pre-1935 | Domainal or nodular nephrite | Turner, 1935 |
| OU1802* | University of Otago / Otago Museum (D 30. 1689) | unknown | Morven, South Canterbury, NZ | pre-1935 | Domainal or nodular nephrite | Turner, 1935 |
| OU1803* | University of Otago / Otago Museum (D 21. 447) | unknown | Warrington, NZ | pre-1935 | Domainal or nodular nephrite | Turner, 1935 |
| OU1804* | University of Otago / Otago Museum (D 33. 1394) | unknown | Otago Beaches, NZ | pre-1935 | Domainal or nodular nephrite | Turner, 1935 |
| OU1805 | University of Otago / Otago Museum (D 33. 1413)^[[1]](#endnote-1)^ | unknown | Taramakau River, NZ | pre-1935 | Domainal or nodular nephrite | Turner, 1935 |
| OU1806* | University of Otago / Otago Museum (D 33. 1395) | unknown | Otago Beaches, NZ | pre-1935 | Domainal or nodular nephrite | Turner, 1935 |
| OU1807* | University of Otago / Otago Museum (D 30. 1739) | unknown | Greenstone Island, S. Canterbury, NZ | pre-1935 | crenulated nephrite | Turner, 1935 |
| OU1808 | University of Otago / Otago Museum (D 33. 1410) | unknown | Westland, NZ | pre-1935 | Foliated semi-nephrite/tremolite schist | Turner, 1935 |
| OU1809* | University of Otago / Otago Museum (D 33. 1380) | unknown | Otago Beaches, NZ | pre-1935 | Domainal or nodular nephrite | Turner, 1935 |
| OU1810* | University of Otago / Otago Museum ( D 33. 1378, D 29. 2377) | unknown | Shag River, NZ | pre-1935 | Domainal or nodular nephrite | Turner, 1935 |
| OU1811* | University of Otago / Otago Museum (D 33. 1406, D 30. 1712) | unknown | Greenstone Island, S. Canterbury, NZ | pre-1935 | Domainal or nodular nephrite | Turner, 1935 |
| OU1812* | University of Otago / Otago Museum (D 33. 1419) | unknown | Taramakau River, NZ | pre-1935 | Foliated semi-nephrite/tremolite schist | Turner, 1935 |
| OU1813* | University of Otago / Otago Museum (D 33. 1409) | unknown | New River, N. Westland, NZ | pre-1935 | Foliated semi-nephrite/tremolite schist | Turner, 1935 |
| OU1814* | University of Otago / Otago Museum (D 30. 1739) | unknown | Greenstone Island, S. Canterbury, NZ | pre-1935 | Foliated semi-nephrite/tremolite schist | Turner, 1935 |
| OU1815* | University of Otago / Otago Museum (D 33. 1400, D 22. 2314) | unknown | Shag River, NZ | pre-1935 | Foliated semi-nephrite/tremolite schist | Turner, 1935 |
| OU1816* | University of Otago / Otago Museum (D 30. 1745) | unknown | Milford, South Canterbury, NZ | pre-1935 | Domainal or nodular nephrite | Turner, 1935 |
| OU1817* | University of Otago / Otago Museum (D 33. 1393, D 30. 1705) | unknown | Greenstone Island, S. Canterbury, NZ | pre-1935 | Foliated semi-nephrite/tremolite schist | Turner, 1935 |
| OU1818* | University of Otago / Otago Museum (D 33. 1377) | unknown | Murdering Beach, Otago, NZ | pre-1935 | Domainal or nodular nephrite | Turner, 1935 |
| OU1819* | University of Otago / Otago Museum (D 21. 496) | unknown | Centre Island, S. Canterbury, NZ | pre-1935 | Domainal or nodular nephrite | Turner, 1935 |
| OU1820* | University of Otago / Otago Museum (D 30. 1742) | unknown | Greenstone Island, S. Canterbury, NZ | pre-1935 | Domainal or nodular nephrite | Turner, 1935 |
| OU1821* | University of Otago / Otago Museum (D 30. 1693) | unknown | Milford, South Canterbury, NZ | pre-1935 | Domainal or nodular nephrite | Turner, 1935 |
| OU1822* | University of Otago / Otago Museum (D 29. 2372, D 33. 1403) | unknown | Shag River, NZ | pre-1935 | Foliated semi-nephrite/tremolite schist | Turner, 1935 |
| OU1852* | University of Otago / Otago Museum (D 19. 230) | unknown | Dart Valley, Western Otago, NZ | pre-1935 | Foliated semi-nephrite/tremolite schist | Turner, 1935 |
| OU2529 | University of Otago | unknown | Caples Valley, NZ | pre-1935 | Domainal or nodular nephrite | Hutton, 1936 |
| OU2542 | University of Otago | unknown | Caples Valley, NZ | pre-1935 | Domainal or nodular nephrite | Hutton, 1936 |
| OU2552 | University of Otago | unknown | Caples Valley, NZ | pre-1935 | Domainal or nodular nephrite | Hutton, 1936 |
| OU2565 | University of Otago | unknown | Caples Valley, NZ | pre-1935 | Foliated semi-nephrite/tremolite schist | Hutton, 1936 |
| OU2567 | University of Otago | unknown | Caples Valley, NZ | pre-1935 | Domainal or nodular nephrite | Hutton, 1936 |
| OU8732 | University of Otago | unknown | Taramakau River, NZ | 1940 | Domainal or nodular nephrite | - |
| OU65889 | University of Otago | A.F. Cooper | Muddy Creek, NW Otago, NZ | 1993 | crenulated nephrite | Cooper 1995 |
| OU65892 | University of Otago | A.F. Cooper | Muddy Creek, NW Otago, NZ | 1993 | crenulated nephrite | Cooper 1995 |

**Supplementary Item 2: Sample descriptions and context**

MR16

Sample MR16 is a sample of *vein nephrite* consisting of a network of tremolite veins cutting a lizardite- and chrysotile-dominated serpentinite shear zone from the Livingstone Fault, New Zealand (Fig 1). The fault is characterised by a serpentinite shear zone tens to hundreds of metres wide that separates the basal ultramafic portions of the Dun Mountain Ophiolite from the continental quartzofeldspathic schists of the Caples Terrane. The sample originates from the metasomatic reaction zone at the contact between the shear zone serpentinite and quartzofeldspatic greyschists at Mount Raddle (Tarling et al., 2019a, 2019b). The tremolite vein networks typically extend 1-2 metres into the serpentinite from the contact between serpentinite and greyschist (Tarling et al., 2019a).

Vein mineralogy: tremolite,

Host rock mineralogy: lizardite, chrysotile, minor: magnetite, Cr-spinel

JC13

Sample JC13 is a sample of *vein nephrite* consisting of a network of tremolite veins in lizardite-chrysotile serpentinite from Jade Cove, California, USA (Fig 2). The outcrop is within Franciscan Complex metasomatized metagreywacke mélange that contains embedded blocks of greenschist, chert and serpentinised ultramafics. The sample originates from a talc-tremolite metasomatic reaction zone at the contact between a serpentinite block in the metagraywacke mélange (Crippen, 1951; King et al., 2003; Hirauchi et al., 2020). The tremolite veins extend away from the metasomatic reaction zone into the serpentinite for ~1 metre and appear heavily folded.

Vein mineralogy: tremolite,

Host rock mineralogy: lizardite, chrysotile, minor: magnetite, Cr-spinel

OU46117

Sample OU46117 is a sample of *crenulated nephrite* from the Pounamu Ultramafic Belt in the Whitcombe River, New Zealand (Fig 3 a-d; Cooper and Reay, 1983; Ireland et al., 1984). The sample is derived from a metasomatic reaction zone at the contact of a lens of meta-serpentinite and the surrounding mélange matrix (Cooper and Reay, 1983). The field occurrence is as white veins of nephrite associated with pyrite, cross-cutting sheared meta-serpentinite. The meta-serpentine occurs as a pod within meta-basalts and quartzofeldspathic schist, and is interpreted on structural and relict (palimpsest) features in the surrounding quartzofeldspathic schists as the core of an antiform. Naturally etched surfaces of the specimen viewed under magnification show the presence of a very finely-laminated, close-spaced foliation that microscopically is defined by the microlithons of a crenulation cleavage.

Nephrite mineralogy: tremolite

OU65872

Sample OU65872 is a sample of *crenulated nephrite* from Muddy Creek, northwest Otago, New Zealand (Fig 3 e-h). At Muddy Creek, a mafic-ultramafic pod is embedded in quartzofeldspathic schist of the Haast Schist (Cooper, 1995). The sample is derived from a tremolite metasomatic zone developed on the faulted margin of a massive tremolite-chlorite-pumpellyite-bearing meta-gabbro pod enclosed in quartzofeldspathic schists. All margins of the pod are heavily slickensided and portions of the metasomatic reaction zone are brecciated. Metamorphic grade is transitional between pumpellyite-actinolite and greenschist facies. The crenulated nephrite sample is a fine-grained schistose rock containing accessory Mg-chlorite, Cr-muscovite and magnetite and possesses a secondary crenulation cleavage.

Nephrite mineralogy: tremolite, minor: fuchsite, clinochlore, magnetite

TW04

Sample TW04 consists of a nephritic *foliated tremolite schist* from a contact reaction zone between serpentinite and metasedimentary schists in a mélange in the Yuli belt, Taiwan (Fig 4). The Yuli belt is a metasedimentary matrix mélange with embedded meta-mafic and -ultramafic blocks (Zhang et al., 2020). The sample comes from a sheared metasomatic reaction zone at the contact between a serpentinised ultramafic block and the surrounding metapelitic mélange. This reaction zone consist of a schistose nephritic tremolite layer up to 0.5 metres thick (Kuo, 2020). The foliated nephrite sample is a fine-grained schistose rock consisting entirety of tremolite.

Nephrite mineralogy: tremolite

HCF2

Sample HCF2 consist of *domainal/nodular nephrite* from the serpentinised ultramafic portion of the Dun Mountain Ophiolite in Hacket Creek, Nelson, New Zealand (Fig 5). The sample was collected in float in Hacket Creek. Numerous nephritic tremolite-bearing metasomatic reaction zones are present in the area at faulted contacts between boudinaged dykes of meta-gabbro and the surrounding sheared serpentinite.

Nephrite mineralogy: tremolite, minor: Cr-spinel

**Supplementary Item 3: Chemical analysis of sample JC13**

The figure and table below show quantitative EDS chemical analyses from tremolite grains in sample JC13. Analyses were taken from matrix tremolite and new tremolite that grew parallel to fold axial planes during formation of an incipient crenulation cleavage.

Figure S1: SEM-BE image of sample JC13, with EDS analysis points indicated (Table S2). The serpentinite matrix is the darker grey (white arrow) and the tremolite is lighter grey (black). The new tremolite grow overgrow and replace the matrix serpentinite.

**
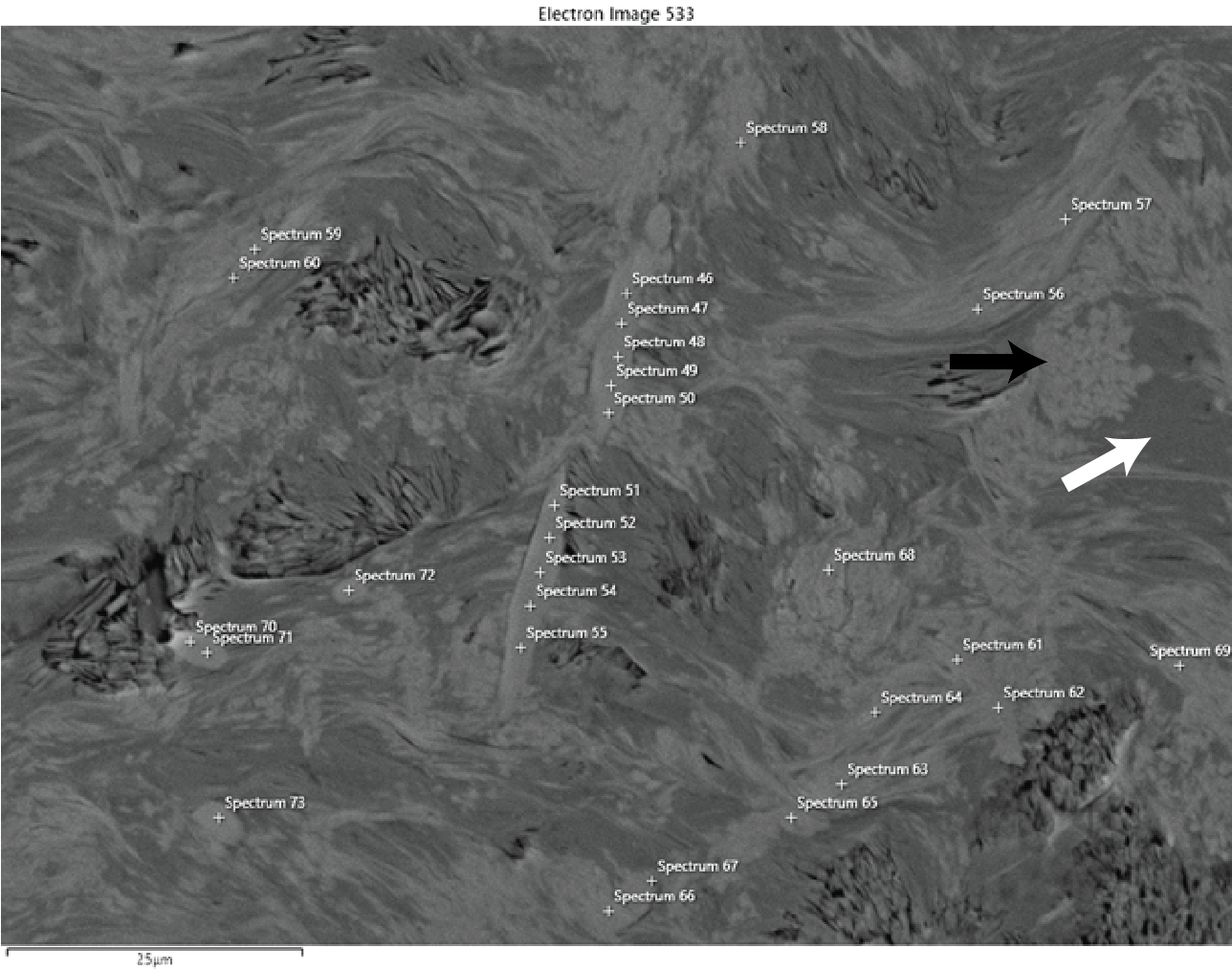
**

Table S2: EDS analyses from tremolite grains in sample JC13. Spectra are labelled on Figure S1. Values are in oxide %. Data points in grey are taken from tremolite crystals that appear to have grown over the main tremolite-serpentinite matrix, including the two relatively large, elongate, and euhedral crystals that are sub-parallel to an axial planar cleavage in JC13. Data points in white are from disseminated tremolite in the folded tremolite-serpentinite matrix.

Newly-grown crystals of tremolite are distinguished chemically from matrix tremolite by having notably higher Ca contents than matrix tremolite.

| Spectrum Label | Na | Mg | Si | Ca | Fe | Total |
| --- | --- | --- | --- | --- | --- | --- |
| Spectrum 46 |  | 22.41 | 57.51 | 12.63 | 4.86 | 97.4 |
| Spectrum 47 |  | 22.77 | 57.76 | 12.6 | 4.32 | 97.45 |
| Spectrum 48 |  | 22.67 | 57.51 | 12.48 | 4.53 | 97.19 |
| Spectrum 49 |  | 22.82 | 57.73 | 12.45 | 4.33 | 97.32 |
| Spectrum 50 |  | 22.58 | 57.32 | 12.49 | 3.99 | 96.37 |
| Spectrum 51 |  | 23.3 | 58.34 | 13.07 | 4.12 | 98.82 |
| Spectrum 52 |  | 23.07 | 58.19 | 13.03 | 4.07 | 98.37 |
| Spectrum 53 |  | 22.83 | 57.73 | 12.85 | 4.28 | 97.7 |
| Spectrum 54 |  | 22.69 | 57.4 | 12.66 | 4.24 | 96.99 |
| Spectrum 55 |  | 22.95 | 57.71 | 12.45 | 3.9 | 97.01 |
| Spectrum 56 | 0.67 | 23.93 | 57.84 | 10.25 | 3.9 | 96.59 |
| Spectrum 57 | 0.49 | 23.91 | 57.65 | 10.25 | 3.87 | 96.17 |
| Spectrum 58 | 0.49 | 23.2 | 57.82 | 11.66 | 4.13 | 97.3 |
| Spectrum 59 | 0.95 | 24.51 | 58.44 | 10.45 | 3.98 | 98.33 |
| Spectrum 60 | 0.57 | 24.35 | 57.98 | 9.36 | 4.1 | 96.36 |
| Spectrum 61 | 1.45 | 24.54 | 58.7 | 10.31 | 3.77 | 98.77 |
| Spectrum 62 | 1.02 | 23.89 | 58.29 | 11.46 | 4.01 | 98.67 |
| Spectrum 63 | 0.98 | 23.42 | 57.68 | 10.64 | 4.04 | 96.76 |
| Spectrum 64 | 0.93 | 24.47 | 58.55 | 10.23 | 4.02 | 98.19 |
| Spectrum 65 | 0.41 | 23.17 | 56.92 | 11.3 | 4.21 | 96.01 |
| Spectrum 66 | 0.87 | 23.34 | 57.17 | 11.7 | 3.89 | 96.97 |
| Spectrum 67 | 0.59 | 24.86 | 58.06 | 8.53 | 3.71 | 95.75 |
| Spectrum 68 | 0.47 | 23.15 | 58.16 | 12 | 4.4 | 98.19 |
| Spectrum 69 |  | 23.53 | 58.27 | 11.55 | 3.95 | 97.3 |
| Spectrum 70 |  | 25.34 | 61.08 | 12.73 | 4.18 | 103.33 |
| Spectrum 71 |  | 22.84 | 57.93 | 12.78 | 4.44 | 97.99 |
| Spectrum 72 |  | 24.13 | 58.58 | 12 | 3.99 | 98.7 |
| Spectrum 73 |  | 23.56 | 57.99 | 11.61 | 4.29 | 97.46 |

**References**

Cooper, A.F., 1995, Nephrite and metagabbro in the Haast Schist at Muddy Creek, northwest Otago, New Zealand: New Zealand Journal of Geology and Geophysics, v. 38, p. 325–332, doi:10.1080/00288306.1995.9514660.

Cooper, A.F., and Reay, A., 1983, Lithology, field relationships, and structure of the Pounamu Ultramafics from the Whitcombe and Hokitika rivers, Westland, New Zealand: New Zealand Journal of Geology and Geophysics, v. 26, p. 359–379, doi:10.1080/00288306.1983.10422254.

Crippen, R.A., 1951, Nephrite jade and associated rocks of the Cape San Martin region, Monterey County, California: California Division of Mines and Geology Special Publication Special Report 10-B,.

Hirauchi, K., Yamamoto, Y., den Hartog, S.A.M., and Niemeijer, A.R., 2020, The role of metasomatic alteration on frictional properties of subduction thrusts: An example from a serpentinite body in the Franciscan Complex, California: Earth and Planetary Science Letters, v. 531, p. 115967.

Hutton, C.O., 1936, Basic and ultrabasic rocks in north-west Otago: Trans. Roy. Soc. NZ, v. 66, p. 231–254.

Ireland, T.R., Reay, A., and Cooper, A.F., 1984, The pounamu ultramafic belt in the diedrich range, Westland, New Zealand: New Zealand Journal of Geology and Geophysics, v. 27, p. 247–256, doi:10.1080/00288306.1984.10422295.

King, R.L., Kohn, M.J., and Eiler, J.M., 2003, Constraints on the petrologic structure of the subduction zone slab-mantle interface from Franciscan Complex exotic ultramafic blocks: Bulletin of the Geological Society of America, v. 115, p. 1097–1109, doi:10.1130/B25255.1.

Kuo, L.-W., 2020, Is the birth of Taiwan nephrite related to plate tectonics?, *in* Taiwan Jade: Past and Present, National Taiwan Museum, p. 100–119.

Tarling, M.S., Smith, S.A.F., and Scott, J.M., 2019a, Fluid overpressure from chemical reactions in serpentinite within the source region of deep episodic tremor: Nature Geoscience, doi:10.1038/s41561-019-0470-z.

Tarling, M.S., Smith, S.A.F., Scott, J.M., Rooney, J.S., Viti, C., and Gordon, K.C., 2019b, The internal structure and composition of a plate boundary-scale serpentinite shear zone: The Livingstone Fault, New Zealand: Solid Earth, p. 1–32, doi:10.5194/se-2019-62.

Turner, F.J., 1935, Geological investigations of the nephrites, serpentines, and related" green stones" used by the Maoris of Otago and South Canterbury: Transactions of the Royal Society of New Zealand, v. 65, p. 187–210.

Zhang, Y., Tsai, C.-H., Froitzheim, N., and Ustaszewski, K., 2020, The Yuli Belt in Taiwan: Part of the suture zone separating Eurasian and Philippine Sea plates.: Terrestrial, Atmospheric & Oceanic Sciences, v. 31.

1. From having retrieved the archival information for this sample from the Otago Museum records, it would appear that the Otago Museum sample number which Turner (1935) provided for this sample contains a mistake. The sample number should be D33.1413 rather than D33.1813, as given by Turner (1935). [↑](#endnote-ref-1)
